# Supplementary material for: Assessing the effectiveness of intervention to prevent plague through community and animal-based survey
Source: PLOS Glob Public Health. 2023 Dec 18;3(12):e0002211. doi: 10.1371/journal.pgph.0002211 (PMC10727364; doi:10.1371/journal.pgph.0002211)
Supplement: Attachment — Submitted filename: PGPH_D_223-00799_Comments on manuscript.pdf [file pgph.0002211.s003.pdf]

Agreed 20/7
Due 30/7.

PLOS Global Public Health

Assessing the effectiveness of intervention to prevent plague through community and animal-based survey

--Manuscript Draft--

**Manuscript Number:**	PGPH-D-23-00799
**Article Type:**	Research Article
**Full Title:**	Assessing the effectiveness of intervention to prevent plague through community and animal-based survey
**Short Title:**	Plague prevention using animal-based survey
**Corresponding Author:**	Soanandrasana Rahelinirina<br>Institut Pasteur de Madagascar<br>Antananarivo, Antananarivo MADAGASCAR
**Order of Authors:**	Soanandrasana Rahelinirina
	Soloandry Rahajandraibe, Master 2
	Sitraka Rakotosamimanana, PhD
	Minoarisoa Rajerison, HDR
**Keywords:**	plague; Incidence; rodent; rural; Madagascar; community
**Abstract:**	Bubonic plague, transmitted by infected flea bites, is the most common form of plague and, left untreated, can progress to the pneumonic form, which is highly contagious. Surveillance focusing on reservoir and vector control is considered to be the main way to prevent plague. Common rodent control methods include the use of rodenticide and snap traps but, in plague context, dispersal of fleas from killed animals poses a serious health threat. Therefore, there is a need for development of strategies to combine reservoir and vector control. The aim of this study was to assess the effects of combination of reservoir and vector control through community-based surveillance. During low plague season, Kartman bait station contained anticoagulant rodenticide with dust insecticide, and live traps were placed indoor and outdoor involved local agent community in active plague foci in Madagascar. Small mammals were counted, identified and killed with their fleas. Effectiveness of control measures was evaluated by comparison of plague incidence two years before and after intervention in two active plague foci with and without intervention using data from the Central Laboratory of Plague. A total of 4,302 small mammals were captured with predominance of the black rat Rattus rattus. Using data on reported cases of plague, our result found the reduction of plague incidence in treated site for two years after treatment. Community-based interventions had in important role in reducing contact between human-rodent-flea. Our study confirms the importance of the animal surveillance during the season of low plague transmission. Combination of reservoir and vector control with community involvement may be effective at reducing the risks of plague spillover to human. The strategy using Kartman bait station indoor with live traps outdoor can be used to refine proactive plague prevention, however, due to the potential development of resistance of fleas and rats to pesticides, overuse should be considered.
**Additional Information:**	
**Question**	**Response**
**Financial Disclosure**<br><br>Enter a financial disclosure statement that describes the sources of funding for the work included in this submission and the role the funder(s) played. This includes	This study was supported financially by the Fondation de Lille (agreement No. 14-001/2016).

Powered by Editorial Manager® and ProduXion Manager® from Aries Systems Corporation

grants and any commercial funding of the work or authors.<br><br>This statement will be typeset if the manuscript is accepted for publication.<br><br>*Please review the submission guidelines and the instructions link below for detailed requirements and guidance.*	
**Competing Interests**<br><br>On behalf of all authors, disclose any competing interests that could be perceived to bias this work.<br><br>This statement will be typeset if the manuscript is accepted for publication.<br><br>*Please review the instructions link below and PLOS Global Public Health's competing interests policy to determine what information must be disclosed at submission.*	The authors declare that they have no competing interests
**Data Availability**<br><br>Before publication, Authors are required to make fully available and without restriction all data underlying their findings. Please see our PLOS Data Policy page for detailed information on this policy.<br><br>A **Data Availability Statement**, detailing where the data can be accessed, is required at first submission. Insert your Data Availability Statement in the box below.<br><br>Please see the data reporting section of our submission guidelines for instructions on what you need to include in your Data Availability Statement.	The datasets used during the current study are available from the corresponding author on reasonable request.

Powered by Editorial Manager® and ProduXion Manager® from Aries Systems Corporation

This statement will be typeset if the manuscript is accepted for publication.<br><br>*PLOS allows rare exemptions to address legal and ethical concerns. If you have legal or ethical restrictions, please use the box below to detail these in full sentences for the Journal team to consider.*	

Powered by Editorial Manager® and ProduXion Manager® from Aries Systems Corporation

Title page

Title: Assessing the effectiveness of intervention to prevent plague through community and animal-based survey

Short title: Plague prevention using animal-based survey

Authors: Soanandrasana Rahelinirina[1*], Soloandry Rahajandraibe[2], Sitraka Rakotosamimanana[1], Minoarisoa Rajerison[1]

Affiliations:

[1]Institut Pasteur de Madagascar, Antananarivo, Madagascar

[2]Ministry of Public Health, Madagascar

***Corresponding author:**

Soanandrasana Rahelinirina,

Institut Pasteur de Madagascar,

BP 1274, Antananarivo 101, Madagascar

Email : rahelinirina@pasteur.mg

Abstract

Bubonic plague, transmitted by infected flea bites, is the most common form of plague and, left untreated, can progress to the pneumonic form, which is highly contagious. Surveillance focusing on reservoir and vector control is considered to be the main way to prevent plague. Common rodent control methods include the use of rodenticide and snap traps but, in plague context, dispersal of fleas from killed animals poses a serious health threat. Therefore, there is a need for development of strategies to combine reservoir and vector control. The aim of this study was to assess the effects of combination of reservoir and vector control through community-based surveillance. During low plague season, Kartman bait station contained anticoagulant rodenticide with dust insecticide, and live traps were placed indoor and outdoor involved local agent community in active plague foci in Madagascar. Small mammals were counted, identified and killed with their fleas. Effectiveness of control measures was evaluated by comparison of plague incidence two years before and after intervention in two active plague foci with and without intervention using data from the Central Laboratory of Plague. A total of 4,302 small mammals were captured with predominance of the black rat *Rattus rattus*. Using data on reported cases of plague, our result found the reduction of plague incidence in treated site for two years after treatment. Community-based interventions had in important role in reducing contact between human-rodent-flea. Our study confirms the importance of the animal surveillance during the season of low plague transmission. Combination of reservoir and vector control with community involvement may be effective at reducing the risks of plague spillover to human. The strategy using Kartman bait station indoor with live traps outdoor can be used to refine proactive plague

prevention, however, due to the potential development of resistance of fleas and rats to pesticides, overuse should be considered.

Introduction

Plague, a zoonotic disease caused by the bacterium *Yersinia pestis*, remains a major public health threat in Madagascar with 80.5% of human case between 2015 - 2018 [1]. Bubonic plague, from an infected flea bite, is the most common form and, left untreated, can progress to the pneumonic form, which is highly contagious between humans.

In Madagascar, the main reservoir of plague is the black rat (*Rattus rattus*), that found everywhere associated with two flea vectors *Xenopsylla cheopis* the oriental rat flea involved throughout the world, but present in Madagascar only on rats living in houses and *Synopsyllus fonquerniei* an endemic flea, parasite of rats living outdoors [2, 3]. In the Central Highlands where most human plague cases occur in Madagascar, plague season runs mainly from October to April [4], but some cases start as early as August [5, 6]. Abundance of rodent population in rural highlands corresponding to the season of low plague transmission as related to crop harvest. however, the abundance of flea vectors populations corresponding to the season of high plague transmission related to temperature and humidity. Considering the rising incidence of plague in rural endemic areas, it is necessary to consider the development of effective measures against plague infection that target all possible routes of transmission and disease persistence.

One of the most common approaches is to use animal-based and community-based interventions to reduce the abundance of rats and their fleas indoor and outdoor to eliminate risks of pathogen spillover to human. Common rodent control measures include the use of acute rodenticide and snap traps [7] but, in the context of plague, dispersal of fleas from killed rats poses a serious health threat.

The aim of this study was to implement an efficient strategy to prevent human plague cases using animal-based surveillance with local community involvement. Evidence of the effectiveness of this strategy was evaluated by comparing morbidity due to plague before and after intervention in rural plague endemic foci.

Materials and Methods

Ethics statement

Before the initiation of the intervention, the local community were informed of the purpose of the study and verbal consent was obtained. All animal capture and handling procedures were done in accordance with directive 2010/63/EU of the European Parliament and of the Council (https://eur-lex.europa.eu/LexUriServ/LexUriServ.do?uri=OJ:L:2010:276:0033:0079:en:PDF) and the guidelines of the American Society of Mammologists for the use of wild animals in research and education [8].

Plague is a notifiable disease and the data used for this study were gathered from the National Plague Control Program (NPCP) in Madagascar with mandatory notification at central level of all suspected plague patient; no ethics approval was required. In addition, information for all cases has been anonymized before data sharing for analysis. Authors had not access to information that could identify individual participants during or after data collection.

Study sites

The study was performed in Tsiroanomandidy district within 3 rural communities (Bemahatazana, Miandrarivo and Ambatolampy) in the Midwest parts of Madagascar (with intervention) and in Ambositra district located in the southern central highlands (without intervention) and distant about 100 km from Tsiroanomandidy (Fig 1). These two districts were chosen for its highest bubonic plague cases reported every year [9,10].

Fig 1. Map of Madagascar showing the studied districts and the limits of plague-endemic foci. The dashed line represents the limits of plague endemic foci

Field experiment

The specific interventions that were assessed included a combination of rodent and vector control coupled with Information-Education-Communication (IEC) activities that were conducted during the low plague season from April to September 2016. Rodent control consisted of both live capture and killing via poison. Kartman bait stations [11] which combine rodent and vector controls and wire mesh live BTS traps [3] were used. Kartman bait station is a wooden box with lid that protect nontarget species and the environment. Interventions were conducted in 112 villages and hamlets, after obtaining the verbal consent of the householders. Per village, 20 to 100 Kartman bait stations containing 15g of bait block anticoagulant rodenticide (Chlorophacinone, BHL, Madagascar) and 150g of insecticide powder fenitrothion 2% (insecticide used in the NPCP) per bait station were set inside households (one bait station per house).

Twenty (20) BTS live traps were set outside, spaced 10m from each other and baited with dried fish and onion. Interventions were conducted for 5 days every month in each village (6 times per village). Kartman bait station and BTS were set in the evening and checked the next morning.

Three hundred (300) fact sheets and 112 booklets that explain the transmission route, symptom, treatment and prevention of plague, which were produced by the Madagascar Ministry of Public Health and other partners, were distributed in each village and posted on a public place. In addition, information, education and communication (IEC) was raised on the rat proofing, impacts of bush fire and waste management. Fifty-nine (59) local community workers and healthcare were recruited and have been trained to conduct the interventions during the 6 months of the study.

Every day, small mammals captured outdoor were counted and identified. Then, animals were humanely killed [8] with their fleas, incinerated and buried. Dead animal discovered indoor cannot be counted, however.

Temporal trend of plague incidence

To evaluate the effectiveness of the intervention, we compared the incidence of human plague between the treatment site (Tsiroanomandidy) and control site (Ambositra). Plague is a disease requiring mandatory notification and all data of notified plague cases in Madagascar are centralized in a database at the Central Plague Laboratory hosted at the Institut Pasteur de Madagascar. The patients were not recruited specially for the study, they are part of mandatory notification of plague case by health centers. Anonymized data from August 2014 to July 2018 (S1 table and S2 table) were used to construct the deviation from the triennial mean of the incidence according to WHO's case definition [12].

Data analysis

Statistical analyses were carried out using Microsoft Excel® and Stata 13® software. The calculation of the trend in deviations from the 5-years mean incidence or incidence fluctuation was adapted from the calculation of the Deviation from decadal mean of incidence (DDMI) performed by Rakotosamimanana *et al.* [10] Based on the estimated population of the district for the study period, we calculated the mean of monthly incidence per year of probable and confirmed human plague cases. Then we calculated the mean incidence for the whole study period. Finally, the trend of the deviations of the mean incidence for the 5 years studied was analysed with a linear regression line of equation $y=ax+b$.

Small mammal abundance was estimated by trap success defined as the ratio of trapped small mammals to the trap night.

Results and discussion

In total, 4,302 small mammals were captured with live traps set outside with 16,800 trap nights during the 6 months of the study with average trap success around the 25%. Two species were captured belonging to *R. rattus* (4130; 96%) and *Suncus murinus* (172; 4%). The Figure 2 shows a fluctuating plague incidences trend over 4 years in both locations. Decreased trend was found in Tsiroanomandidy after the intervention and one year later (Fig 2A). Furthermore, plague cases started later in the plague season, starting in October instead of August. However, in Ambositra, an increased trend was observed across the same time period (Fig 2B).

Fig 2: Deviation from the quadrennial mean of the incidence (per 100,000 inhabitants) from August 2014 to July 2018 in Tsiroanomandidy with intervention (A) and Ambositra without

intervention (B). The grey curve represents the deviation from the quadrennial mean of the incidence (DQMI); the black curve represents the quarterly moving averages of the DQMI; the dashed line is a linear regression line showing the trend of the DQMI for the period study.

Our study demonstrates that rodent control both indoor and outdoor coupled with IEC activities during the season of low plague transmission can be used to reduce incidence of human plague through a decrease in reservoir and vector densities. Previous study showed that in Tsiroanomandidy, human cases occur every year and in 2014, *Y. pestis* was isolated from *R. rattus* [13]. This study shows that *R. rattus* was still the predominant small mammals in rural zone. In Madagascar, low plague season corresponding to cold and dry season and the abundance of rodent is highest related to the crop harvest while the flea abundance is low due to the cold temperature and low humidity [3]. The use of Kartman bait station during low plague season decrease the risk of dispersion of fleas than during the high plague season which the abundance of fleas is high. The use of anticoagulant rodenticide has the advantage of killing rodents in 4 days post ingestion that leaves time for the insecticide to work and kill the fleas before the rodents and thus prevent their dispersion.

The previous study shown that using intervention only inside home was not sufficient to reduce rodent population abundance due to the recolonization of outdoor rat [14]. Rodent control would need both indoor and outdoor regularly because rodent density re-invade rapidly. To undertake a successful rodent and vector control, accurate data on the biology and behavior of rodent species and good knowledge of the local environmental conditions are necessary prior the study [15]. Outdoor rodenticide treatment is not advised due to the environmental (ecological) condition and non-target species.

Kartman bait station is benefit as delivery of rodenticide and insecticide simultaneously in reducing rodent and flea population. However, whilst insecticide and rodenticide have been shown to be effective, their sustained use risks exacerbating the development of resistance [16,17]. Choosing an appropriate rodenticide and insecticide in referring to national program is one of the important prerequisites to render the intervention effective. In Madagascar, interventions using animal-based surveillance are rarely implemented apart from outbreak investigation. Using Kartman bait station during epidemic situations is permitted by using only insecticide without rodenticide because the aim should be to eliminate the fleas before they bite humans. Rodent and flea controls should be implemented before the beginning of the human plague season at village level. Healthcare workers alone are not sufficient to solve the problem in prevention of plague but the involvement of local community is necessary. In Tsiroanomandidy, bushfire is commonly used to produce green growth for livestock and to prepare agricultural field at the end of dry season (August and September). This period coincided with the end of low plague season. Fire has an impact in the habitat of rodent predator such as bird or snake, can affect climate change that promote the stages of development of fleas and may allow rodent to displace and increase contact between rodent and human. Educating local communities needs to balance advantage and disadvantage of fire [18]. Radio and television play a role in the communication and education of people but given the low resources of the population, they are not widely accessible. Therefore, community-based sensitization and fact sheets illustrated in local language are the best way to communicate disease risk. Due to the potential development of resistance of fleas to insecticides [16], the regular assessment of the status of fleas to insecticide must be realized. As surveillance of rodent and flea populations for the entire endemic plague foci is costly, using this strategy is a very simple way and at a low cost by monitoring rodent both indoor and outdoor.

Which strategy. Need to define here

The limitations of this study are the lack of data for flea index which could be useful to complete the effectiveness of the use of insecticide Kartman bait station and the difficulty to measure the number of rodent activities inside the Kartman bait station (bait eaten and footprints on insecticide powder). However, the feeling of local population proved the reduction of rodent abundance during the use of Kartman bait station in households.

Conclusions

Our study confirms the importance of the animal surveillance during low plague transmission to reduce indoor and outdoor small mammal abundance. We conclude that prioritize prevention is a very effective way, given the high financial costs when outbreaks occurred.

The strategy using Kartman bait station indoor with live traps outdoor can be used to refine proactive plague prevention, however, due to the potential development of resistance of fleas to insecticides, the use of various insecticides is recommended. Our finding can serve as a guide for determining when and how rodent control should be done in order to reduce plague in rural zone. Long-term actions focusing on education, hygiene around villages in reducing refuse and cover, food storage and rat proofing are the strategies that could lead to plague diminution and the decentralization of health services can improve the access to health care.

Authors' contributions

M Rajerison, S Rahelinirina: conceptualization, supervision, visualization, project administration

S Rahajandraibe, S Rahelinirina, S Rakotosamimanana: Data curation & Formal analysis

M Rajerison: Funding acquisition and Resources

M Rajerison, S Rahajandraibe, S Rahelinirina: Investigation

M Rajerison, S Rahelinirina, S Rakotosamimanana: Methodology

S Rahajandraibe, S Rakotosamimanana: Software, analysis

S Rahelinirina: writing – Original draft preparation

M Rajerison, S Rahajandraibe, S Rahelinirina, S Rakotosamimanana: Writing – Review & Editing

Acknowledgments

I would like to thank all those that participated in this study especially the community health workers and health care agents in the district of Tsiroanomandidy, the population participating in Bemahatazana, Miandrarivo and Ambatolampy, the staff of Plague Unit and Parfait Andriniaina. This study was supported financially by the Fondation de Lille and technically by Madagascar Ministry of Public Health and Institut Pasteur de Madagascar (agreement No. 14-001/2016).

References

1. Bertherat E. Plague around the world in 2019. Wkly Epidemiol Rec. 2019;94(25):289-92.
2. Brygoo ER. Epidémiologie de la peste à Madagascar. Arch Inst Pasteur Madagascar. 1966;35(39–11):9–149

3. Rahelinirina S, Duplantier JM, Ratovonjato J, Ramilijaona O, Ratsimba M, Rahalison L. Study on the Movement of *Rattus rattus* and Evaluation of the Plague Dispersion in Madagascar. Vector-Borne Zoonotic Dis. 2010;10(1):77-84.

4. Chanteau S, Ratsifasoamanana L, Rasoamanana B, Rahalison L, Randriambelosoa J, Roux J. and Rabeson D. Plague, a reemerging disease in Madagascar. Emerg Infect Dis. 1998; 4:101-103.

5. Ramasindrazana B, Andrianaivoarimanana V, Rakotondramanga JM, Birdsell DN, Ratsitorahina M, Rajerison M. Pneumonic plague transmission, Moramanga, Madagascar. Emerg Infect Dis. 2015;23(3):521-24.

6. Randremanana R, Andrianaivoarimanana V, Nikolay B, Ramasindrazana B, Paireau J, Bosch QA, et al. Epidemiological characteristics of urban plague epidemic in Madagascar, August-November 2017: an outbreak report. Lancet Infect Dis. 2019;19(5):537-45.

7. Constant NL, Swanepoel LH, Williams ST, Soarimalala V, Goodman SM, Massawe AT, et al. Comparative assessment on rodent impacts and cultural perceptions of ecologically based rodent management in 3 Afro-Malagasy farming regions. Integr Zool. 2020;15(6):578-94.

8. Sikes RS and the Animal Care and Use Committee of the American Society of Mammalogists. Guidelines of the American Society of Mammalogists for the use of wild mammals in research and education. J Mammal. 2016;97(3): 663-688.

9. Andrianaivoarimanana V, Piola P, Wagner DM, Rakotomanana F, Maheriniaina V, Andrianalimanana S, et al. Trends of Human Plague, Madagascar, 1998-2016. Emerg Infect Dis. 2019;25(2):220-28.

10. Rakotosamimanana S, Kassie D, Taglioni F, Ramamonjisoa J, Rakotomanana F, Rajerison M. A decade of plague in Madagascar: a description of two hotspot districts. BMC Public Health. 2021; doi 10.1186/s12889-021-11061-8

11. Ratovonjato J, Duchemin JB, Duplantier JM, Rahelinirina S, Soares JL, Rahalison L, et al. Lutte contre la peste à Madagascar : évaluation de l'efficacité des boîtes de Kartman en milieu urbain. Arch Inst Pasteur Madagascar. 2003;69 (1&2):41–45.

12. World Health Organization. International meeting on preventing and controlling plague: the old calamity still has a future. Wkly Epidemiol Rec. 2006;81:278–84.

13. Rakotoarisoa A, Ramihangihajason T, Ramarokoto C, Rahelinirina S, Halm A, Piola P, et al. Bubonic plague outbreak Investigation in the endemic district of Tsiroanomandidy – Madagascar, October 2014. J Case Rep Stud. 2017;4 (6) ISSN 2348-9820

14. Rahelinirina S, Scobie K, Ramasindrazana B, Andrianaivoarimanana V, Rasoamalala F, Randriantseheno LN, Rakotoniaina JS, Gorgé O, Lambin X, Valade V, Telfer S and Rajerison M. Rodent control to fight plague: field assessment of methods based on rat density reduction. Integ. Zool. 2021 doi: 10.1111/1749-4877.12529.

15. Singleton GR, Brown PR, Jacob J. Ecologically-based rodent management: its effectiveness in cropping systems in South-East Asia. NJAS. 2004;52(2):163-171.

16. Miarinjara A, Boyer S. Current Perspectives on Plague Vector Control in Madagascar: Susceptibility Status of Xenopsylla cheopis to 12 Insecticides. PLOS Neglect Trop Dis. 2016;10: e0004414. doi:10.1371/journal.pntd.0004414

17. Boyle M. Case of apparent resistance of Rattus norvegicus Berkenhout to anticoagulant poisons. Nature. 1960; 188: 517–525.

18. Mistry J, Berardi A. Bridging indigenous and scientific knowledge. Science. 2016;352(6291):1274-5.

Supporting information

S1 Table

S2 Table

[illegible]

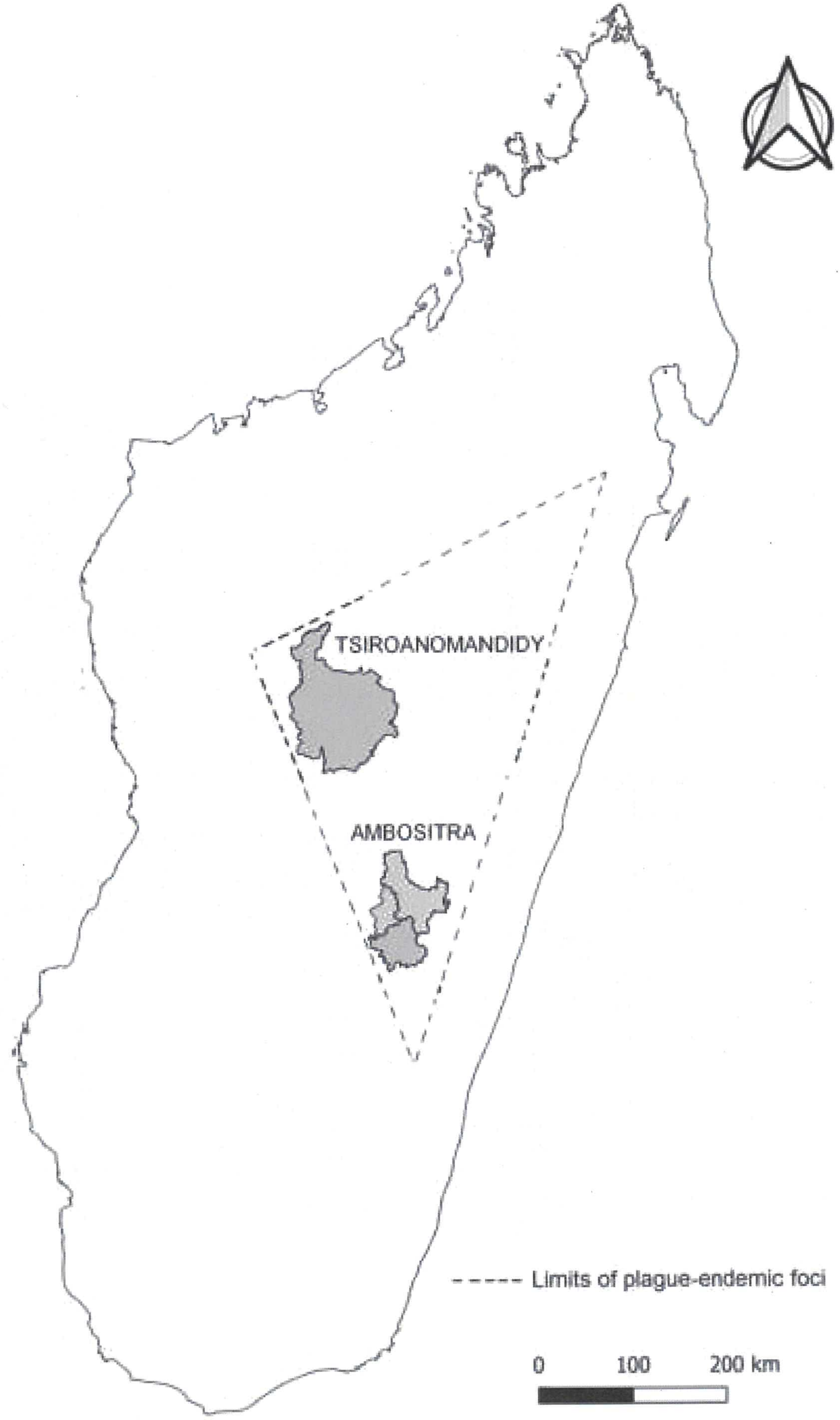
TSIROANOMANDIDY
AMBOSITRA
Limits of plague-endemic foci
0
100
200 km

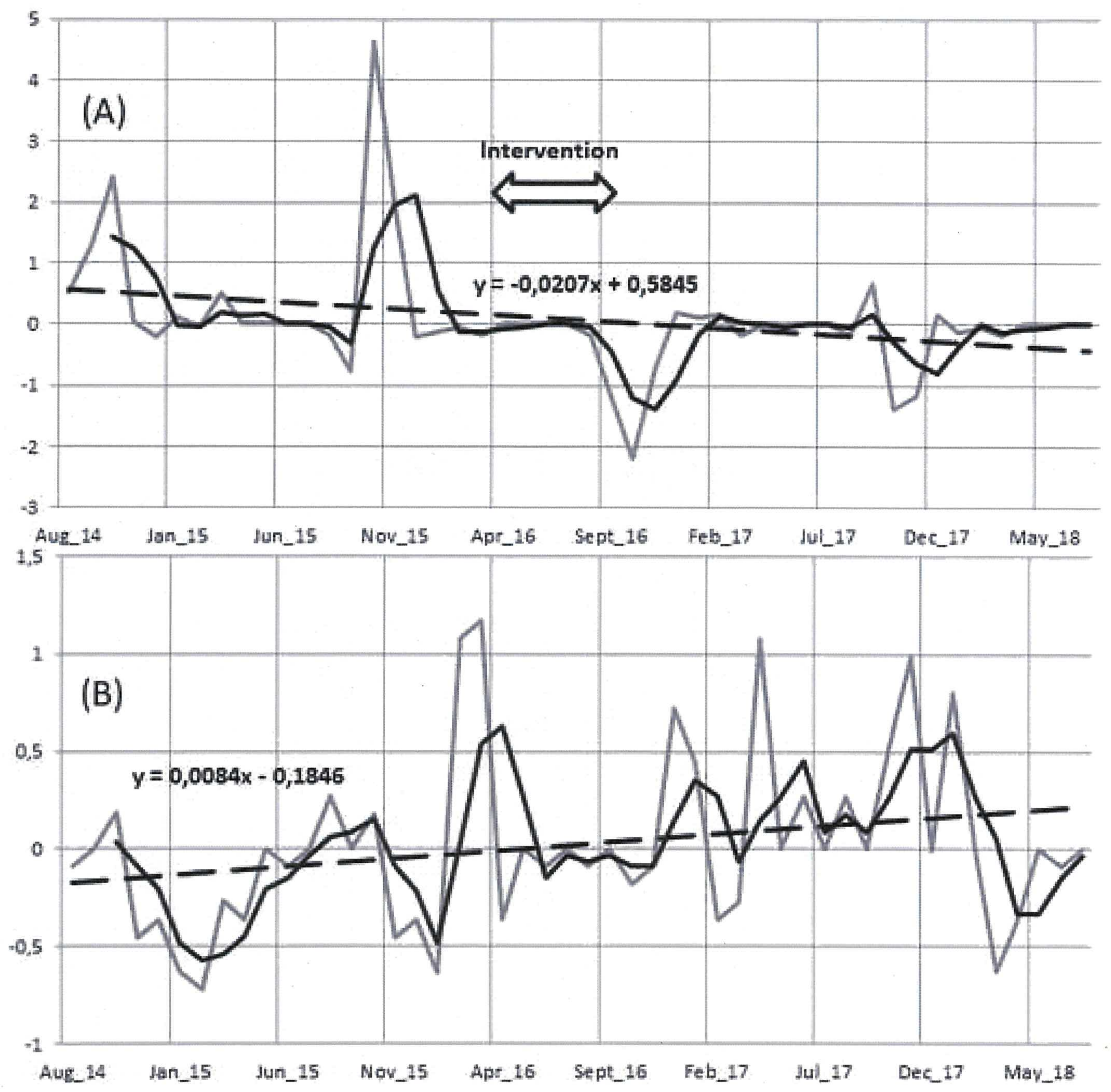
(A)
Intervention
y = -0,0207x + 0,5845
5
4
3
2
1
0
-1
-2
-3
Aug_14
Jan_15
Jun_15
Nov_15
Apr_16
Sept_16
Feb_17
Jul_17
Dec_17
May_18
(B)
y = 0,0084x - 0,1846
1,5
1
0,5
0
-0,5
-1
Aug_14
Jan_15
Jun_15
Nov_15
Apr_16
Sept_16
Feb_17
Jul_17
Dec_17
May_18

Click here to access/download
Supporting Information
S1 table.pdf

Click here to access/download
Supporting Information
S2 table.pdf
