# Supplementary material for: Assessing the effectiveness of intervention to prevent plague through community and animal-based survey
Source: PLOS Glob Public Health. 2023 Dec 18;3(12):e0002211. doi: 10.1371/journal.pgph.0002211 (PMC10727364; doi:10.1371/journal.pgph.0002211)
Supplement: Attachment — Submitted filename: Response to Reviewers.docx [file pgph.0002211.s004.docx]

**Reviewers’ comments and authors’ responses**

On behalf of all co-authors, we highly appreciate the reviewers’ insightful and helpful comments on our manuscript as well as the precious time spent in reviewing our paper about “Assessing the effectiveness of intervention to prevent plague through community and animal-based survey”.

**Reviewer #1**

The authors bring in the manuscript results from Assessing the effectiveness of an intervention to prevent plague through community and animal-based surveys. The approach is quite complete. The surveillance the authors report on has valuable information to share. It is good to report surveillance results in known endemic regions to determine risks to plague. Therefore, the publication is important

**Thanks a lot for your time and expertise.**

**Reviewer #2**

The authors demonstrate the importance of animal surveillance in areas of low plague prevalence. And two ways to refine proactive plague prevention, indoor and outdoor, are proposed. This strategy is economic and effective. The author’s results and exploration of prevention are also very meaningful for reducing plague epidemics and preventing plague epidemics in others parts of the world. Since the author’s work was carried out in 2016, it is recommended to add some progress of related work in recent years to the discussion.

Thank you for your positive feedbacks and your suggestion. Accordingly, we have provided some pieces of information about more recent actions in Madagascar (see Discussion).

**Reviewer #3**

The authors have presented only detailed analysis of the human data. There is very little analysis of the rodent trapping data which would be more informative to a wider audience interested in the rodent side of the study as well as the human incidence of plague infection. Details of the rodent trapping data are not available. English expression requires considerable improvement. See attached pdf.

Thank you for pointing these out. We fully agree with your comment. In order to address the rodent issue, we have now added table and figure which summarises rodent-associated data available. In addition, some results were provided in the results, and were further discussed in the Discussion section.

Thank you for checking the English wording which was greatly improved the manuscript**.**
